# Supplementary material for: Superionic Ionic Conductor Discovery via Multiscale Topological Learning
Source: J Am Chem Soc. 2025 Jun 5;147(24):20888–98. doi: 10.1021/jacs.5c04828 (PMC12186519; doi:10.1021/jacs.5c04828)
Supplement: Supplementary file 1 [file ja5c04828_si_001.pdf]

Supplementary Information

Superionic Ionic Conductor Discovery via Multiscale Topological Learning

Dong Chen[1,2,†], Bingxu Wang[1,†], Shunning Li[1], Wentao Zhang[1], Kai Yang[1], Yongli Song[1], Guo-Wei Wei[2,4,*] and Feng Pan[1,*]

[1]School of Advanced Materials, Peking University, Shenzhen Graduate School, Shenzhen 518055, China

[2]Department of Mathematics, Michigan State University, MI, 48824, USA

[3]Department of Electrical and Computer Engineering, Michigan State University, MI 48824, USA

[4]Department of Biochemistry and Molecular Biology, Michigan State University, MI 48824, USA

[†]These authors contributed equally to this work

[*]Corresponding author: weig@msu.edu, panfeng@pkusz.edu.cn

We started from the ICSD-2019 database, from which structures synthesized under high-pressure and low-pressure conditions were first removed. Subsequently, all non-inorganic entries, invalid structures, and structures with a total partial occupancy exceeding 1 were filtered out. We then identified and extracted all Li-containing structures. Additionally, seven invalid structures that failed to be parsed using pymatgen were manually corrected. After incorporating these corrections, we compiled a set of all valid Li-containing structures, initially totaling 2728 entries.

To focus on purely ionic lithium conductors, we excluded compounds containing transition metals (such as Fe, Mn, Ni, Co, Ti, Mo, and V), resulting in 2725 structures. Three problematic structures (ICSD IDs: 182025, 84765, and 84091) were further excluded due to abnormal features, leaving 2725 structures. Subsequently, two additional entries were removed: 95178.cif (identified as an intermetallic compound) and 418765.cif (an alloy with a minor amount of nitrogen), reducing the dataset to 2723 structures. Due to computational complexity, structures 153218.cif and 87409.cif were also excluded, leaving 2597 structures. Five structures lacking any cation elements (ICSD IDs: 103787, 107824, 51966, 53095, and 634548) were then removed, resulting in 2592 valid entries. Finally, at the feature computation stage, two structures (153218.cif and 87409.cif) were again excluded due to extreme structural complexity, leading to a final dataset of 2590 structures for subsequent modeling and screening.

Table S1. The number of compounds that passed each steps of the high-throughput screening of crystal structural framework

**Screening Step**	**Number of Compositions**
Remove Duplicate	2590
Filter Connection Radius	1443
Filter Servive Betti1	770
Clustering Methods	339
Final screening	43

Table S2. List of known LSICs

**ICSD-IDs**	**Compositions**	**Superionic conductor**
100169	$Li_{14}Zn(GeO_4)_4$	LISCISON
157654	$Li_7P_3S_{11}$	$Li_7P_3S_{11}$
163297	$Li_{1.2}(Al_{0.2}Ge_{0.2}Ti_{1.6})\ (PO_4)_3$	NASICON
188887	$Li_{10}GeP_2S_{12}$	LGPS
422259	$Li_7La_3Zr_2O_{12}$	LLZO
50422	$Li_{2.64}(Sc_{0.9}Ti_{0.1})_2(PO_4)_3$	$Li_3A_2(PO_4)_3$
35018	Li_3PS_4	Li-P-S
92200	Li_4GeS_4	Li-Ge-S
174202	$Li_7La_3Hf_2O_{12}$	Garnet
191542	$Li_{6.46}Al_{0.08}1La_3Zr_2O_{12}$	Garnet
185539	$Li_{6.06}Al_{0.20}1La_3Zr_2O_{12}$	Garnet
185540	$Li_{6.06}Al_{0.20}1La_3Zr_2O_{12}$	Garnet
238693	$La_3Li_{5.8}Zn_{0.6}Zr_2O_{12}$	Garnet
238692	$La_3Li_{5.8}Zn_{0.6}Zr_2O_{12}$	Garnet
238690	$Al_{0.24}La_3Li_{6.28}Zr_2O_{12}$	Garnet
238689	$Al_{0.24}La_3Li_{6.28}Zr_2O_{12}$	Garnet
195182	$(Li_{6.26}Al_{0.24})La_3Zr_2O_{12}$	Garnet
69076	$La_3Li_5Sb_2O_{12}$	Garnet
174612	$Li_6(CaLa_2)Sb_2O_{12}$	Garnet
174615	$Li_{6.4}(Ca_{1.4}La_{1.6})Sb_2O_{12}$	Garnet
174617	$Li_{6.4}(Ca_{1.4}La_{1.6})Sb_2O_{12}$	Garnet
158373	$Li_6SrLa_2Bi_2O_{12}$	
161342	$Li_5La_3(Sb_2O_{12})$	Garnet
161343	$Li_5La_3(Sb_2O_{12})$	Garnet
161389	$Li_5La_3(SbO_6)_2$	Garnet
163914	$Li_7La_3Sn_2O_{12}$	Garnet
182034	$Li_7La_3Sn_2O_{12}$	Garnet
182035	$Li_{2.25}H_{4.75}La_3Sn_2O_{12}$	Garnet
182036	$Li_{4.59}H_{2.41}La_3Sn_2O_{12}$	Garnet
245641	$Li_5La_3(SbO_6)_2$	Garnet

245934	$Li_6SrLa_2Sb_2O_{12}$	Garnet
245935	$Li_{6.4}Sr_{1.4}La_{1.6}Sb_2O_{12}$	Garnet
246817	$Li_7La_3Zr_2O_{12}$,	Garnet
261302	$Li_7La_3Zr_2O_{12}$,	Garnet
183684	$Li_7La_3Zr_2O_{12}$,	LLZO
183685	$Li_7La_3Zr_2O_{12}$,	Garnet
184230	$Li_7La_3(ZrO_6)_2$	Garnet
191528	$Li_7La_3Zr_2O_{12}$,	Garnet
183607	$Li_7La_3Zr_2O_{12}$,	Garnet
238687	$Li_7La_3Zr_2O_{12}$,	Garnet
238686	$Li_7La_3Zr_2O_{12}$,	Garnet
65025	$Li_2Mg_2(SO_4)_3$	$Li_3A_2(PO_4)_3$
62300	$Li_3Sc_2(PO_4)_3$	$Li_3A_2(PO_4)_3$
91113	$Li_{2.72}Ti_2(PO_4)_3$	$Li_3A_2(PO_4)_3$
202539	$Li_{2.68}(Ti_{1.86}Al_{0.14})(PO_4)_3$,	NASICON
94029	$(Li_{0.72}Na_{0.88})(Ti_{1.4}Al_{0.6})(PO_4)_3$	NASICON
94030	$(Li_{1.09}Na_{0.71})(Ti_{1.2}Al_{0.8})(PO_4)_3$	NASICON
94031	$(Li_{1.45}Na_{0.45})(Ti_{1.1}Al_{0.9})(PO_4)_3$	NASICON
163299	$Li_{1.2}(Al_{0.2}Ge_{0.4}Ti_{1.4})(PO_4)_3$	NASICON
163300	$Li_{1.4}(Al_{0.4}Ge_{0.4}Ti_{1.2})(PO_4)_3$	NASICON
163301	$Li_{1.6}(Al_{0.6}Ge_{0.4}Ti)(PO_4)_3$	NASICON
163302	$Li_{1.2}(Al_{0.2}Ge_{0.6}Ti_{1.2})(PO_4)_3$	NASICON
163303	$Li_{1.4}(Al_{0.4}Ge_{0.6}Ti)(PO_4)_3$	NASICON
163304	$Li_{1.6}(Al_{0.6}Ge_{0.6}Ti_{0.8})(PO_4)_3$	NASICON
163305	$Li_3Ti_2(PO_4)_3$, $Li_{2.93}Ti_2(PO_4)_3$	NASICON
193036	$Li_{2.93}Ti_2(PO_4)_3$	NASICON
193037	$Li_{2.8}Ti_2(PO_4)_3$,	NASICON
193032	$Li_{1.2}(Al_{0.2}Ge_{0.6}Ti_{1.2})(PO_4)_3$	NASICON
193038	$Li_3Ti_2(PO_4)_3$	NASICON
193033	$Li_{1.6}(Al_{0.6}Ge_{0.4}Ti)(PO_4)_3$	NASICON
92250	$LiZr_2(PO_4)_3$	$Li_3A_2(PO_4)_3$
97658	$Li_2Ti_2(PO_4)_3$	NASICON
97659	$Li_{2.96}(Sc_{0.9}Zr_{0.1})_2(PO_4)_3$	NASICON
97660	$LiZr_2(PO_4)_3$	NASICON
191891	$Li_{1.15}(Y_{0.15}Zr_{1.85})(PO_4)_3$	NASICON
245527	$Li_{0.7}La_{0.1}Zr_2(PO_4)_3$	NASICON
257190	$Li_{1.12}Ti_{1.88}In_{0.12}(PO_4)_3$	NASICON
20208	$Li_3(PO_4)$	LISICON
25816	$Li_3Zn_{0.5}(GeO_4)$	LISICON
34221	$Li_{2.5}Ga_{0.5}GeO_4$	LISICON
50058	Li3PO4	Li-P-O
79427	$Li_{3.5}Zn_{.25}GeO_4$	LISICON
77095	$Li_{14}Zn(GeO_4)_4$	LISICON

8280	Li_2MgGeO_4	LISICON
65643	$Li_{2.5}Ga_{0.5}GeO_4$	LISICON
78513	$Li_3Zn_{.5}GeO_4$	LISICON
100167	$Li_{3.5}Zn_{.25}GeO_4$	LISICON
100169	$Li_{6.6}SiPO_8$	LISICON
202631	Li_3PO_4	LISICON
202632	$Li_{2.5}Ga_{0.5}GeO_4$	LISICON
166547	Li_2MgGeO_4	LISICON
238601	$Li_{2.5}Ga_{0.5}GeO_4$	LISICON
290831	Li_4SnS_4	Li-Ge-S
290832	Li_4SnS_4	Li-Ge-S
50578	Li_4GeS_4	Li-Ge-S
92200	Li_4GeS_4	Li-Ge-S
95649	Li_4GeS_4	Li-Ge-S
188886	$Li_{10}(GeP_2S_{12})$	LGPS
188887	$Li_{10}(SnP_2S_{12})$	LGPS
193755	$Li_{10.35}((Ge_{1.35}P_{1.65})S_{12})$	LGPS
193947	$Li_{10.35}Si_{1.35}P_{1.65}S_{12}$	LGPS
241439	$Li_{9.81}Sn_{0.81}P_{2.19}S_{12}$	LGPS
252036	$Li_{10}(Ge_{0.416}Sn_{0.584})P_2S_{12}$	LGPS
252037	$Li_{10}(Ge_{0.776}Sn_{0.224})P_2S_{12}$	LGPS
252038	$Li_{10.35}(Sn_{0.27}Si_{1.08})$, $P_{1.65}S_{12}$	LGPS
252039	$Li_{10.35}(Sn_{0.27}Si_{1.08})$, $P_{1.65}S_{12}$	LGPS
252040	$Li_{10.5}(Sn_{0.2}Si_{0.8})_{1.5}P_{1.5}S_{12}$	LGPS
252041	$Li_{10.2}(Sn_{0.2}Si_{0.8})_{1.2}P_{1.8}S_{12}$	LGPS

Table S3. Lithium-containing compounds that passed the high-throughput screening of crystal structural framework but rejected by other considerations

**ICSD-IDs**	**Compositions**	**Reasons**
19024, 27007, 27008, 30964, 33809, 33810, 36532, 56443, 60363, 61086, 61087, 65127, 78059, 84240, 96224, 239794, 243972, 253007, 257739, 281011, 415121, 416926, 416927, 416928, 416929, 417653, 421259, 421549, 423635, 432026, 19023, 67238, 92571, 421548, 260280	Li_2CaGeO_4, $Na_3Li_3Sc_2F_{12}$, $Na_3Li_3In_2F_{12}$, KLiO, $Cs_2Li_2(SiO_4)$, $Cs_2Li_2(TiO_4)$, $Cs_2Li_2(GeO_4)$, LiSrP, $Na_3Li_3Ti_2F_{12}$, $Rb_2Li_2SiO_4$, $Rb_2Li_2GeO_4$, $LiGaGeO_4$, $Li_2(TiSiO_5)$, $Li(Al_7B_4O_{17})$, $LiSr(GaN_2)$, $LiSc(SeO_3)_2$, $LiCsCO_3$, $LiGaGe_2Se_6$, $LiGaGeO_4$, Li_2PbGeS_4, $Li_2(TeSe_3)$, $Li_3Na_3(InF_6)_2$, $Li_6Y_3(PS_4)_5$, $Li_4Sr_3(Si_2N_6)$, $SrLi_2N_4Si_2$, $CsLi_2Cl_3$,	Bad Li network

	$Li_3Y(BO_3)_2$, Li_2CaSiO_4, $LiAlGeO_4$, $KLi(CO_3)$, $CaLi_2N_4Si_2$, $Li_2Zn_3(P_2O_7)_2$	
610785	Li_3As	Binary compounds
90147	$Cs_{14}Li_{22.72}(Li_{18.4}Si_{72}O_{172})(D_2O)_{6.16}$	Containing D_2O
88334	$Li_6(P_6O_{18})(H_2O)_{9.86}$	Containing H
6269, 67925, 171366, 62586, 428547	$Li_{0.91}(Al_{0.91}Si_{1.09}O_4)(H_2O)_{0.98}$, $Li_8(HPO_4)(Be_6P_6O_{24})(H_2O)$, $K_{2.6}Li_{5.4}(Li_4Si_{16}O_{38})(H_2O)_{4.3}$, $Li_4(P_4O_{12})(H_2O)_5$, $(Li_2(H_2O)_7)Se$	Containing H_2O
251594	$Li_6RbB_2O_6F$	Containing O and F
50992	$Li(Y_{0.95}Eu_{0.05})O_2$	Containing Y, Eu
432591	Li_4PN_3	High-pressure hynthesis. The product is sensitive to traces of air and moisture.
161763, 250297, 50612	$Li_2Ga_2GeS_6$, $Li_2Ti(GeO_4)O$, $Li_3Sc_2(PO_4)_3$, $Li_2(BAlO_4)$	ehull>50emV
188886, 188887, 193755, 193947, 241439, 252036, 252037, 252038, 252039, 252040, 252041, 255747, 255748, 255749, 257946, 257947, 257948	$Li_{10}(GeP_2S_{12})$, $Li_{10}(SnP_2S_{12})$, $Li_{10.35}((Ge_{1.35}P_{1.65})S_{12})$, $Li_{10.35}Si_{1.35}P_{1.65}S_{12}$, $Li_{9.81}Sn_{0.81}P_{2.19}S_{12}$, $Li_{10}(Ge_{0.416}Sn_{0.584})P_2S_{12}$, $Li_{10}(Ge_{0.776}Sn_{0.224})P_2S_{12}$, $Li_{10.35}(Sn_{0.27}Si_{1.08})$, $P_{1.65}S_{12}$, $Li_{10.5}(Sn_{0.2}Si_{0.8})_{1.5}P_{1.5}S_{12}$, $Li_{10.2}(Sn_{0.2}Si_{0.8})_{1.2}P_{1.8}S_{12}$	LGPS
161342, 161343, 161389, 163914, 174202, 174612, 174613, 174614, 174615, 174616, 174617, 182034, 182035, 182036, 183607, 183685, 184230, 185539, 185540, 191528, 191542, 195182, 238686, 238687, 238689, 238690, 238692, 238693, 241475, 245641, 245934, 245935, 246817, 253056, 261302, 430571, 430575, 430576, 430602, 69076	$Li_5La_3(Sb_2O_{12})$, $Li_6(SrLa_2)(Sb_2O_{12})$, $Li_5La_3(SbO_6)_2$, $Li_7La_3Sn_2O_{12}$, $Li_7La_3Hf_2O_{12}$, $Li_6(CaLa_2)Sb_2O_{12}$, $Li_{6.4}(Ca_{1.4}La_{1.6})Sb_2O_{12}$, $Li_7La_3Sn_2O_{12}$, $Li_{2.25}H_{4.75}La_3Sn_2O_{12}$, $Li_{4.59}H_{2.41}La_3Sn_2O_{12}$, $Li_7La_3Zr_2O_{12}$, $Li_7La_3(ZrO_6)_2$, $Li_{6.06}Al_{0.20}La_3Zr_2O_{12}$, $Li_7La_3Zr_2O_{12}$, $Li_{6.46}Al_{0.081}La_3Zr_2O_{12}$, $(Li_{6.26}Al_{0.24})La_3Zr_2O_{12}$, $La_3Li_7Zr_2O_{12}$, $Al_{0.24}La_3Li_{6.28}Zr_2O_{12}$, $La_3Li_{5.8}Zn_{0.6}Zr_2O_{12}$, $Li_{51.2}Al_{1.6}La_{24}Zr_{16}O_{96}$, $Li_5La_3(SbO_6)_2$, $Li_5La_3(SbO_6)_2$,	Garnet

	$Li_{6.4}Sr_{1.4}La_{1.6}Sb_2O_{12}$, $Li_7La_3Zr_2O_{12}$, $Li_7La_3Hf_2O_{12}$, $Li_{5.91}Al_{0.216}La_{2.95}Zr_2O_{12}$, $Al_{0.11}Ga_{0.08}La_{2.94}Li_{6.85}Zr_2O_{12}$, $Al_{0.19}Ga_{0.05}La_{2.88}Li_{5.55}O_{12}Zr_2$, $Li_{6.55}Ga_{0.05}La_{2.91}Zr_2O_{12}$, $La_3Li_5Sb_2O_{12}$	
23017, 38350, 50421, 68494, 253896, 431368, 431573, 642303, 35405, 65445, 255464, 40247, 55665, 72100, 35290, 65126, 73223, 79098, 8372, 169799, 26817, 249872, 249873, 254974	$Li_4Zn(PO_4)_2$, Li_4PbO_4, $Li_{3.2}Sc_2(PO_4)_3$, $Li_{10}(Zn_4O_9)$, $Li_{10}Si_2P_6$, Li_2SiP_2, $LiSbS_2$, $Rb_2Li_{14}(Pb_3O_{14})$, $K_2Li_{14}(Zr_3O_{14})$, $Li_4Zn(PO_4)_2$, Li_6TeO_6, $LiAlSiO_4$, Li_3GaSiO_5, $LiAlSi_2O_6$, Li_3AsS_3, $K_2Li_3GaO_4$, $LiAlGeO_4$, Li_2ZnBr_4, $Li_5La_3Bi_2O_{12}$, $Li_{2.2}Zn_{0.8}P_2O_7$, Li_2CdGeS_4, Li_2CdSnS_4, $Li_2CdGeSe_4$	Duplicate structure
47143, 47144, 47145, 428002, 14364, 67702, 67703, 95407, 239962, 254107, 30276	$Li_{3.175}(SiO_4)_{0.7}(SO_4)_{0.3}$, $Li_{3.17}Si_{0.7}S_{0.3}O_4$, $Li_{3.4}Si_{0.7}S_{0.3}O_4$, $Li_5(B(SO_4)_4)$, $LiNa(SO_4)$, $Li_4(SO_4)_2$, $LiNaSO_4$, Li_2SO_4	Containing SO_4^{2-}
25308, 42062, 42068, 171497, 419577, 426692, 429881	Li_9Ge_4, LiGe, $Li_{11}Ge_6$, $Li_8Zn_2Ge_3$, $Ge_{12}Li_7$, $Li_{18}Na_2Ge_{17}$, Li_3NaGe_2	Germanide, Ge unusual valence state
26298, 80593, 255465, 255466, 255468, 264522, 78514, 153218, 255467	$(Sr_7Li_4Si_{10})_{0.1429}$, $Li_{2.5}Al_{0.5}(SiO_4)$, $Li_4Zn(PO_4)_2$, $Li_{3.7}Zn_{0.7}Ga_{0.3}(PO_4)_2$, $Li_{3.5}Zn_{0.5}Ga_{0.5}(PO_4)_2$, $Hf_2La_3(Li_{6.74}Al_{0.08})O_{12}$, $Li_{2.42}Ga_{0.58}GeO_4$, $Li_{2.46}Al_{0.18}(BO_3)$, $Li_{3.7}Zn_{0.7}Ga_{0.3}(PO_4)_2$	Li sharing sites with: Si, Al, Zn, Ga
50422, 62300, 65025, 71277, 91113, 92250	$Li_{2.64}(Sc_{0.9}Ti_{0.1})_2(PO_4)_3$, $Li_3Sc_2(PO_4)_3$, $Li_2Mg_2(SO_4)_3$, $Li_{2.72}Ti_2(PO_4)_3$, $LiZr_2(PO_4)_3$	$Li_3A_2(PO_4)_3$
1037, 16229	Li_5AlO_4	Li-Al-O
433369	Li_5AlS_4	Li-Al-S
165579, 413238	$Li(ClO_4)$	Li-Cl-O
31050, 100403	$Li_6Ge_2O_7$, $Li_2(GeO_3)$	Li-Ge-O
50578, 92200, 95649, 290831, 290832	Li_4GeS_4, Li_4SnS_4	Li-Ge-S
638, 50058, 79426, 85714, 246859, 248414, 257439	LiPO3, Li3PO4, $Li2_{.88}PO_{3.73}N_{0.14}$, $Li_6P_6O_{18}$, $Li_4P_2O_7$	Li-P-O
35018, 157654, 180318,	Li_3PS_4, $Li_7(P_3S_{11})$, $Li_4P_2S_6$	Li-P-S

242170		
48106	Li_2SeO_4	Li-Se-O
8280, 20031, 20208, 25816, 34221, 65643, 77095, 78513, 79427, 100167, 100169, 166547, 202631, 202632, 238601, 257440	Li_2MgGeO_4, Li_2CdGeO_4, $Li_3(PO_4)$, $Li_3Zn_{0.5}(GeO_4)$, $Li_{2.5}Ga_{0.5}GeO_4$, $Li_{14}Zn(GeO_4)_4$, $Li_2Mg(GeO_4)$, $Li_3Zn_{.5}GeO_4$, $Li_{3.5}Zn_{.25}GeO_4$, $Li_{6.6}SiPO_8$, Li_3PO_4	LISICON
65176	$Li_8O_2SiO_4$	Li-Si-O
58, 2512, 23723, 59243, 153806, 431359	Li_2SO_4, $Li_4P_2O_7$, $Li_2S_5O_{16}$	Li-S-O
415120	Li_2TeS_3	Li-Te-S
16219	Li_5TlO_4	Li-Tl-O
75164	Li_4TiO_4	Li-Ti-O
59646, 59647, 99395, 99396, 99397, 99398, 99399, 99402, 236888	$(La_{0.62}Li_{0.16})(TiO_3)$, $(Li_{0.18}La_{0.122})La_{0.483}(TiO_3)$, $(Li_{0.18}La_{0.119})La_{0.483}(TiO_3)$, $(Li_{0.18}La_{0.118})La_{0.484}(TiO_3)$, $(Li_{0.18}La_{0.123})La_{0.485}(TiO_3)$, $(Li_{0.18}La_{0.118})La_{0.492}(TiO_3)$, $(Li_{0.18}La_{0.121})La_{0.482}(TiO_3)$, $La_{4.44}Li_{2.66}Ti_8O_{24}$	LLTO
183684, 238685, 238688, 238691, 422259, 83652	$Li_7La_3Zr_2O_{12}$, $La_3Li_7Zr_2O_{12}$, $Al_{0.24}La_3Li_{6.28}Zr_2O_{12}$, $La_3Li_{5.8}Zn_{0.6}Zr_2O_{12}$, $Li_7La_3Zr_2O_{12}$	LLZO
254406	$LiGaGe_2S_6$	Low Melting Point
1384, 243929, 264211, 391200, 421083, 426103	$Li(SO_3F)$, $Li_{24}Sr_{12}(Si_{24}N_{47}O)F$, $Li_{40.70}Y_{3.30}Ca_{5.70}(Li_2Si_{30}N_{59})O_2F$, $Li_{5.88}H_{4.5}(P_{12}O_2N_{22})Cl_{2.556}$, $Li_6(PS_4)(SI)$, $Li_9Mg_3(PO_4)_4F_3$	Multiple anions
63018, 94029, 94030, 94031, 95980, 95981, 163297, 163298, 163299, 163300, 163301, 163302, 163303, 163304, 163305, 193032, 193033, 193034, 193035, 193036, 193037, 193038, 193039, 193040, 193041, 202538, 202539, 257190, 261825, 50423, 97658, 97659, 97660, 191891, 245527	$Li_{1.15}Ti_{1.85}In_{0.15}P_3O_{12}$, $(Li_{0.72}Na_{0.88})(Ti_{1.4}Al_{0.6})(PO_4)_3$, $(Li_{1.09}Na_{0.71})(Ti_{1.2}Al_{0.8})(PO_4)_3$, $(Li_{1.45}Na_{0.45})(Ti_{1.1}Al_{0.9})(PO_4)_3$, $Li_{3.18}Ti_2(PO_4)_3$, $Li_{1.2}(Al_{0.2}Ge_{0.2}Ti_{1.6})(PO_4)_3$, $Li_{1.4}(Al_{0.4}Ge_{0.2}Ti_{1.4})(PO_4)_3$, $Li_{1.6}(Al_{0.6}Ge_{0.2}Ti_{1.2})(PO_4)_3$, $Li_{1.2}(Al_{0.2}Ge_{0.4}Ti_{1.4})(PO_4)_3$, $Li_{1.4}(Al_{0.4}Ge_{0.4}Ti_{1.2})(PO_4)_3$, $Li_{1.6}(Al_{0.6}Ge_{0.4}Ti)(PO_4)_3$, $Li_{1.2}(Al_{0.2}Ge_{0.6}Ti_{1.2})(PO_4)_3$, $Li_{1.4}(Al_{0.4}Ge_{0.6}Ti)(PO_4)_3$, $Li_{1.6}(Al_{0.6}Ge_{0.6}Ti_{0.8})(PO_4)_3$,	NASICON

	$Li_3Ti_2(PO_4)_3$, $Li_{2.93}Ti_2(PO_4)_3$, $Li_{2.8}Ti_2(PO_4)_3$, $Li_3(Ti_{1.82}Al_{0.18})(PO_4)_3$, $Li_3(Ti_{1.79}Al_{0.21})(PO_4)_3$, $Li_3(Ti_{1.78}Al_{0.22})(PO_4)_3$, $Li_3(Ti_{1.83}Al_{0.17})(PO_4)_3$, $Li_{2.8}(Ti_{1.82}Al_{0.18})(PO_4)_3$, $Li_{2.68}(Ti_{1.86}Al_{0.14})(PO_4)_3$, $Li_{1.12}Ti_{1.88}In_{0.12}(PO_4)_3$, $Li_{1.15}Ti_{1.85}In_{.15}(PO_4)_3$, $Li_{1.3}Al_{0.3}Ti_{1.7}(PO_4)_3$, $Li_2Ti_2(PO_4)_3$, $Li_{2.96}(Sc_{0.9}Zr_{0.1})_2(PO_4)_3$, $LiZr_2(PO_4)_3$, $Li_{1.15}(Y_{0.15}Zr_{1.85})(PO_4)_3$, $Li_{0.7}La_{0.1}Zr_2(PO_4)_3$	
432318	$Li_{141}N_{126}B_9P_{42}$	Number atoms > 500, further calculations due
60774, 100465, 642222	Li3P7, LiP	P unusual valence state
78836, 107304	Li_5NBr_2, LiCaN	Redox reactions without the participation of Li
100020	Li_2Sb	Sb unusual valence state
67359, 193768, 264695	LiNaSe, Li_4SnSe_4, $Li_2ZnSe_2O_6$	Selenide
431316	$Li_{18}N_{16}P_6$	sigma,348k,7.7E-8 to 1.46E-6; E_a=48.3kJ/mol
24146, 41023, 87408, 281277, 430497, 642372	Li_2Si, Li_8MgSi_6, $Li_{2.35}Si$, $Al_{3.39}Li_{14.61}Si_6$, $Li_{11.56}Na_{0.44}Si_7$	Silicide
182961	$(Li_2(Ti_3O_7))_{1.143}$	Ternary oxides containing Ti
250066	$Li_{3.05}Ge_{0.31}P_{0.69}O_4$	Excluded from further calculations due to the large number of fractional occupied ions in the unit cell
26472, 409657	LiAs, As_7Li_3	As unusual valence state
158373, 238600, 255750, 420126	$Li_6SrLa_2Bi_2O_{12}$, $Li_{6.5}Si_{0.5}P_{1.5}O_8$, $Li_{10}SnP_2S_{12}$, Li_2SiN_2	Very high conductive after doped

Table S4. Lithium-containing compounds that passed the high-throughput screening of crystal structural framework

**ICSD-IDs**	**Compositions**	**Structure Type**
1411	$Li_4(PbO_4)$	Li_4PbO_4
2319	Li_2BeSiO_4	$Li_2BeSiO_4(oS_{32})$
4317	Li_2TeO_3	Li_2TeO_3

6169	$Li_6Al_2(BO_3)_4$	$Li_3AlB_2O_6$
8237	$Li_2Zn(SiO_4)$	Li_2ZnSiO_4
9987	$Li_6Ga_2(BO_3)_4$	$Li_3AlB_2O_6$
15631	$Li_7(SbO_6)$	
23634	$Li_{10}(Zn_4O_9)$	
26297	$Li_6(TeO_6)$	Li_6TeO_6
26817	$LiAl(Si_2O_6)$	$LiAlSi_2O_6$
35250	$K_2Li_{14}(Pb_3O_{14})$	$K_2Li_{14}Pb_3O_4$
37071	$Li_3Na_2(GaO_4)$	$Li_3Na_2GaO_4$
38324	Li_3KGeO_4	$CsNa_3TiO_4$
40245	Li_3BiO_3	
40457	$LiSbS_2$	$AgAsS_2$
50950	$LiZn(PO_4)$	$LiZnPO_4$
59381	$Li_3(AsS_3)$	
59640	$Li_4Zn(PO_4)_2$	$Li_4O_8P_2Zn$
61204	$Na_4Li_5(BO_3)_3$	
67991	$Li_{14}Be_5B(BO_3)_9$	
69967	$NaLi_2(PO_4)$	Li_3PO_4
71035	$KLi_6(BiO_6)$	KLi_6IrO_6
72840	$Li_6K(BiO_6)$	
74864	$CsKNa_2Li_8(Li(SiO_4))_4$	$CsKNa_2Li_8(LiSiO_4)_4$
74865	$RbNa_3Li_8(Li(SiO_4))_4$	$CsKNa_2Li_8(LiSiO_4)_4$
78326	$Li_{10}(Si_2PbO_{10})$	
78819	$Li_{10}N_3Br$	
86184	$LiZn(AsO_4)$	$LiGaSiO_4$
86458	$Li_3Sc_2(PO_4)_3$	$Li_3Fe_2(PO_4)_3$
87596	$Li_2(TiO)(SiO_4)$	Li_2TiSiO_5
92708	$Li(AlSiO_4)$	$LiGaSiO_4$
95972	$Li_2(MgSiO_4)$	Li_2ZnSiO_4
202115	$NaLi_3(SiO_4)$	$NaLi_3SiO_4$
202116	$NaLi_3GeO_4$	$NaLi_3SiO_4$
241234	$Li_3Sc(BO_3)_2$	
252215	Li_3AlSiO_5	$AlFeO_3$
253895	$Li_{10}Si_2P_6$	
257740	$LiGa_{0.5}Al_{0.5}GeO_4$	$LiGaSiO_4$
262642	$In_2Li_2SiS_6$	Cd_4GeS_6
262643	$In_2Li_2GeS_6$	Cd_4GeS_6
402341	$LiBeN$	
413355	$(LiI)_2(Li_3(SbS_3))$	
424834	Li_3SbS_3	
424835	Li_3AsS_3	
431367	Li_2SiP_2	

Table S5. Ionic conductivity at 800 K, 1000 K, 1200 K, and 1400 K and room temperature (300K) and activation barrier of Lithium-containing compounds that passed the high-throughput screening of crystal structural framework

ICSD-IDs	Compositions	Ionic conductivity					Activation barrier	SE_{E_a}
		800K	1000K	1200K	1400K	300K		
1411	$Li_4(PbO_4)$	1.146	5.196e-1	4.740e+1	3.473e+3	4.341e-15	1.345	0.576
2319	Li_2BeSiO_4	7.644e-1	5.898e-1	1.579e-1	2.377e-1	7.724e+1	-0.148	1.100
4317	Li_2TeO_3	1.782	6.974e-10	1.542e+2	8.987e+2	6.879e-22	1.729	3.176
6169	$Li_6Al_2(BO_3)_4$	2.396e+1	1.490e+1	3.967e+1	5.567e-10	3.515e+34	-2.935	2.402
8237	$Li_2Zn(SiO_4)$	2.739e-1	7.158e-10	8.013e-2	5.113e-10	1.708e+19	-1.973	2.542
9987	$Li_6Ga_2(BO_3)_4$	1.266	4.612e+1	1.698e+2	8.764e+1	1.291e-8	0.826	0.241
15631	$Li_7(SbO_6)$	7.079e+2	1.119e+3	1.491e+3	1.642e+3	7.634e	0.228	0.012
23634	$Li_{10}(Zn_4O_9)$	5.251	3.960e+1	9.849e+1	1.359e+2	4.694e-6	0.624	0.069
26297	$Li_6(TeO_6)$	2.822	8.745e-1	5.879e-1	9.491e-10	7.908e+30	-2.71	1.907
26817	$LiAl(Si_2O_6)$	8.973e+1	1.302e+2	2.644e+2	2.456e+2	3.194e-1	0.272	0.042
35250	$K_2Li_{14}(Pb_3O_{14})$	1.122	5.584e-1	4.608e+1	2.170e+2	3.291e-11	0.998	0.398
37071	$Li_3Na_2(GaO_4)$	5.986e-1	2.907e-1	1.648e-1	6.053e-10	5.059e+28	-2.567	1.847
38324	Li_3KGeO_4	2.183	8.618e-10	7.182e-10	7.627e+2	1.359e-4	0.056	3.791
40245	Li_3BiO_3	6.923e+1	7.249e+2	7.665e+2	1.669e+3	2.327e-4	0.573	0.115
40457	$LiSbS_2$	5.444e+1	2.734e+1	1.143e+2	2.578e+2	1.897e-2	0.349	0.175
50950	$LiZn(PO_4)$	6.880e-1	8.750e-1	1.108e-1	2.157e-1	1.600e+2	-0.176	1.175
59381	$Li_3(AsS_3)$	1.056e+3	1.975e+3	2.058e+3	3.407e+3	5.220	0.260	0.035
59640	$Li_4Zn(PO_4)_2$	1.220e+2	4.183e+1	1.568e+2	9.221e+2	1.320e-2	0.387	0.263
61204	$Na_4Li_5(BO_3)_3$	4.649e-1	7.186e-10	3.186e+1	2.645e+2	6.732e-22	1.693	2.993
67991	$Li_{14}Be_5B(BO_3)_9$	9.244e-1	5.546e-1	7.154e-10	1.144e+2	6.494e+5	-0.591	2.970
69967	$NaLi_2(PO_4)$	9.218e-1	1.588e-2	2.441	3.572	1.516e-5	0.429	0.607
71035	$KLi_6(BiO_6)$	2.294e+1	2.622e+1	1.322e+1	1.336e+2	6.162e-2	0.272	0.227
72840	$Li_6K(BiO_6)$	1.329	8.498e-1	5.465e-1	1.205e+2	4.792e-7	0.611	0.543
74864	$CsKNa_2Li_8(Li(SiO_4))_4$	2.780e-1	1.509e+1	3.397	1.002	1.885e-3	0.297	0.423
74865	$RbNa_3Li_8(Li(SiO_4))_4$	5.855e-1	5.761e-1	3.648e-1	2.216e-1	7.972	-0.061	1.057
78326	$Li_{10}(Si_2PbO_{10})$	5.724e+2	7.318e+2	1.398e+3	1.823e+3	1.453	0.283	0.038
78819	$Li_{10}N_3Br$	3.298e+2	1.192e+3	2.010e+3	2.815e+3	2.597e-2	0.435	0.036
86184	$LiZn(AsO_4)$	1.117e-1	1.390e-1	8.598e-2	2.360e-10	5.009e+26	-2.436	1.917
86458	$Li_3Sc_2(PO_4)_3$	2.132e+2	6.799e+2	5.602e+2	7.723e+2	7.987e-1	0.279	0.074
87596	$Li_2(TiO)(SiO_4)$	4.022e-01	6.572e-10	5.476e-10	4.694e-10	1.072e+32	-3.206	1.321
92708	$Li(AlSiO_4)$	3.725e-3	3.332e-2	3.937e-2	3.443e-2	2.504e-7	0.455	0.146
95972	$Li_2(MgSiO_4)$	6.694e-2	5.208e-1	6.012e-1	2.268e-1	1.255e-4	0.323	0.207
202115	$NaLi_3(SiO_4)$	4.066e-1	9.919e-10	8.266e-10	7.085e-10	2.478e+31	-3.142	1.294
202116	$NaLi_3GeO_4$	1.426e-1	1.235e-1	3.641e-1	6.537e-10	6.402e+24	-2.255	1.948

241234	$Li_3Sc(BO_3)_2$	9.860e-10	3.113e-1	6.573e-10	3.655e+1	1.586e-35	2.586	1.386
252215	Li_3AlSiO_5	4.912e-1	1.963e-1	7.578e-10	6.495e-10	1.655e+40	-3.703	2.902
253895	$Li_{10}Si_2P_6$	4.076	2.398	1.538e+1	7.410e+1	9.315e-06	0.549	0.236
257740	$LiGa_{0.5}Al_{0.5}GeO_4$	4.407e-10	3.525e-10	2.938e-10	2.518e-10	1.175e-09	0.0	0.005
262642	$In_2Li_2SiS_6$	7.233e-1	3.057	1.545e+2	5.927e+1	2.546e-10	0.934	0.275
262643	$In_2Li_2GeS_6$	2.952e-10	7.809	2.034e+2	2.910e+2	2.814e-56	4.589	1.401
402341	LiBeN	3.459e-1	2.325e-1	8.006e-2	9.694e-2	3.598e+1	-0.15	1.071
413355	$(LiI)_2(Li_3(SbS_3))$	3.776e+2	1.049e+3	1.983e+3	2.650e+3	5.336e-2	0.408	0.019
424834	Li_3SbS_3	8.335e-10	4.754e+2	1.546e+3	1.635e+3	4.350e-56	4.654	1.728
424835	Li_3AsS_3	1.216e+3	1.468e+3	2.479e+3	3.150e+3	7.378	0.247	0.032
431367	Li_2SiP_2	3.286e+2	6.504e+2	1.392e+3	2.373e+3	4.137e-2	0.408	0.030

Table S6. Lithium-containing compounds that passed the threshold of activation barriers

**ICSD-IDs**	**Compositions**	**Structure Type**	**Activation barrier**	**ESW**
9987	$Li_6Ga_2(BO_3)_4$	$Li_3AlB_2O_6$	0.826	1.654
15631	$Li_7(SbO_6)$		0.228	1.137
23634	$Li_{10}(Zn_4O_9)$		0.627	1.405
26817	$LiAl(Si_2O_6)$	$LiAlSi_2O_6$	0.272	0
35250	$K_2Li_{14}(Pb_3O_{14})$	$K_2Li_{14}Pb_3O_4$	0.998	0.553
40245	Li_3BiO_3		0.573	0.977
40457	$LiSbS_2$	$AgAsS_2$	0.349	0.160
59381	$Li_3(AsS_3)$		0.260	0.203
59640	$Li_4Zn(PO_4)_2$	$Li_4O_8P_2Zn$	0.387	1.772
69967	$NaLi_2(PO_4)$	Li_3PO_4	0.429	3.190
71035	$KLi_6(BiO_6)$	KLi_6IrO_6	0.272	1.064
72840	$Li_6K(BiO_6)$		0.611	1.063
74864	$CsKNa_2Li_8(Li(SiO_4))_4$	$CsKNa_2Li_8(LiSiO_4)_4$	0.297	2.382
78326	$Li_{10}(Si_2PbO_{10})$		0.283	0
78819	$Li_{10}N_3Br$		0.435	0.53
86458	$Li_3Sc_2(PO_4)_3$	$Li_3Fe_2(PO_4)_3$	0.279	0
92708	$Li(AlSiO_4)$	$LiGaSiO_4$	0.455	2.667
95972	$Li_2(MgSiO_4)$	Li_2ZnSiO_4	0.323	2.739
253895	$Li_{10}Si_2P_6$		0.549	0.373
262642	$In_2Li_2SiS_6$	Cd_4GeS_6	0.934	0.755
413355	$(LiI)_2(Li_3(SbS_3))$		0.408	0.325
424835	Li_3AsS_3		0.247	0.204
431367	Li_2SiP_2		0.408	0.427

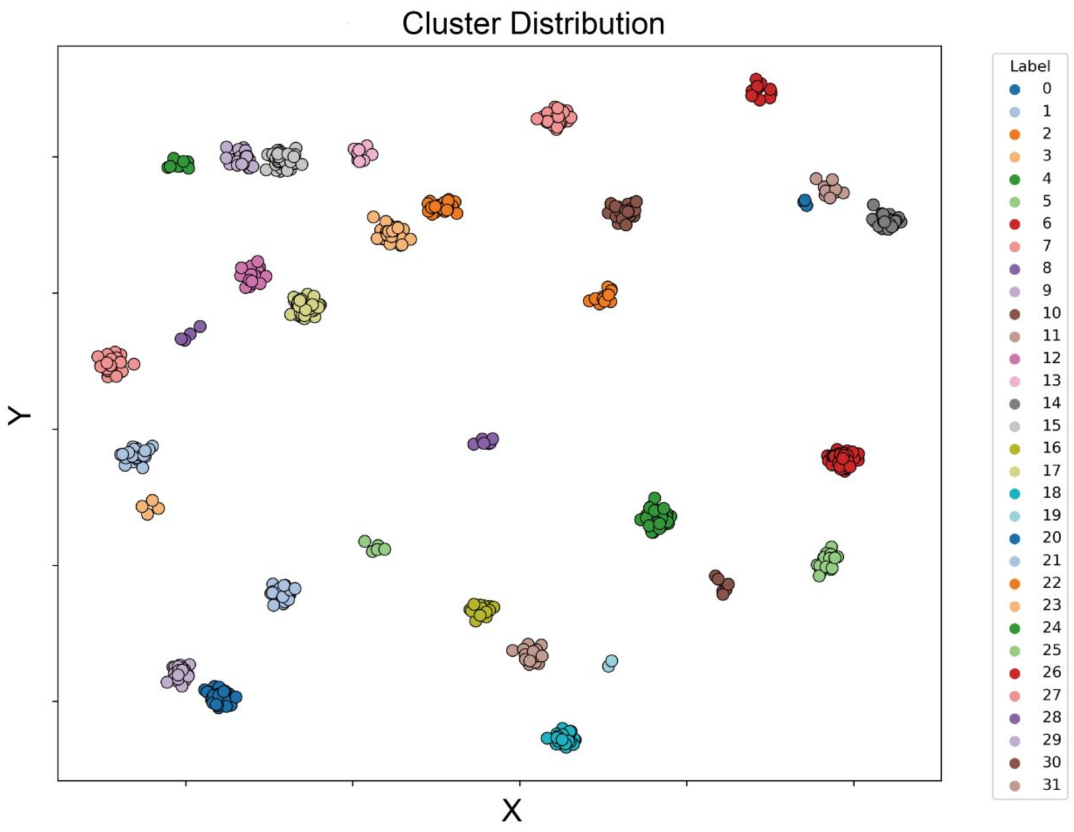

Figure S1. Visualization of the topological feature space derived from multiscale topological learning. Each point represents a lithium-containing compound. The wide distribution of predicted candidates across the feature space indicates substantial structural and compositional diversity, suggesting that these materials are not trivial variants of known systems.

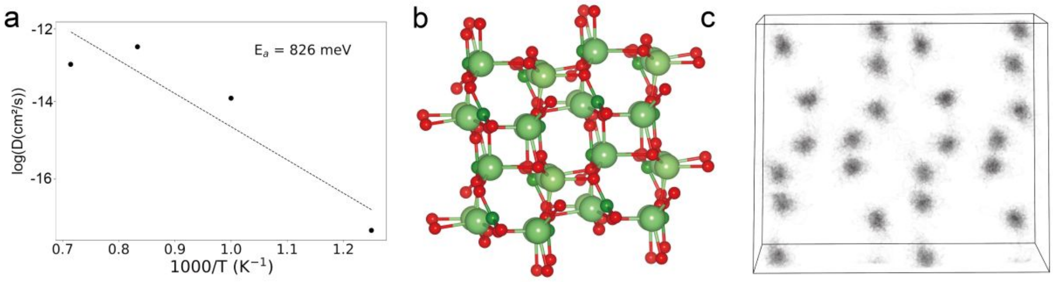

Figure S2. AIMD simulations of $Li_6Ga_2(BO_3)_4$. a) Arrhenius plot of lithium-ion diffusion coefficient. b) The structure schematic. c) Isosurface of lithium-ion probability density from the 800-1400K AIMD simulation.

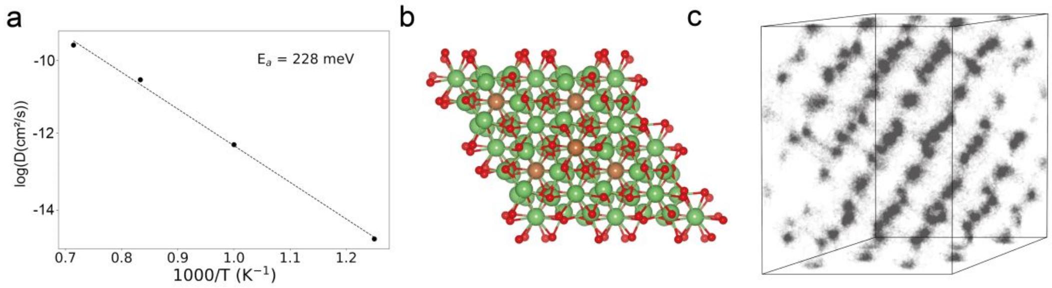

Figure S3. AIMD simulations of Li_7SbO_6. a) Arrhenius plot of lithium-ion diffusion coefficient. b) The structure schematic. c) Isosurface of lithium-ion probability density from the 800-1400K AIMD simulation.

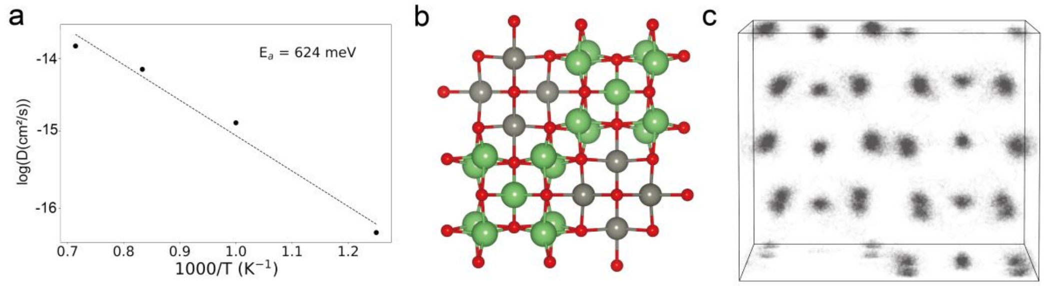

Figure S4. AIMD simulations of $Li_{10}Zn_4O_9$. a) Arrhenius plot of lithium-ion diffusion coefficient. b) The structure schematic. c) Isosurface of lithium-ion probability density from the 800-1400K AIMD simulation.

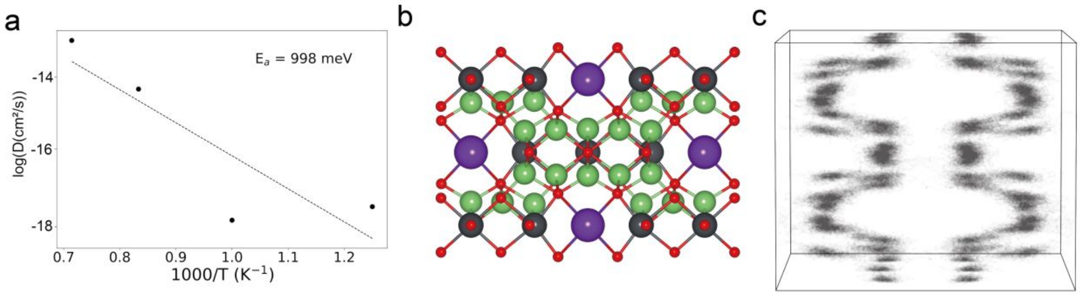

Figure S5. AIMD simulations of $K_2Li_{14}Pb_3O_{14}$. a) Arrhenius plot of lithium-ion diffusion coefficient. b) The structure schematic. c) Isosurface of lithium-ion probability density from the 800-1400K AIMD simulation.

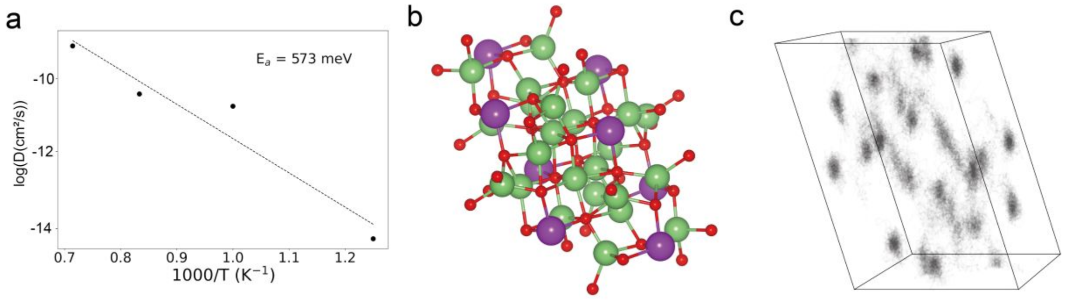

Figure S6. AIMD simulations of Li_3BiO_3. a) Arrhenius plot of lithium-ion diffusion coefficient. b) The structure schematic. c) Isosurface of lithium-ion probability density from the 800-1400K AIMD simulation.

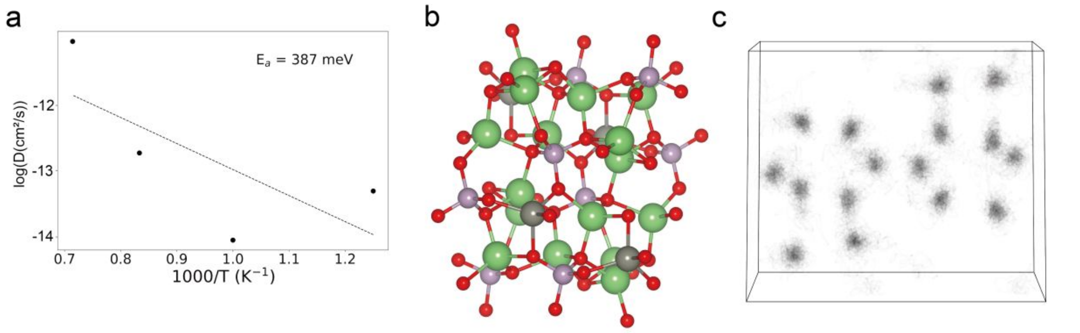

Figure S7. AIMD simulations of $Li_4Zn(PO_4)_2$. a) Arrhenius plot of lithium-ion diffusion coefficient. b) The structure schematic. c) Isosurface of lithium-ion probability density from the 800-1400K AIMD simulation.

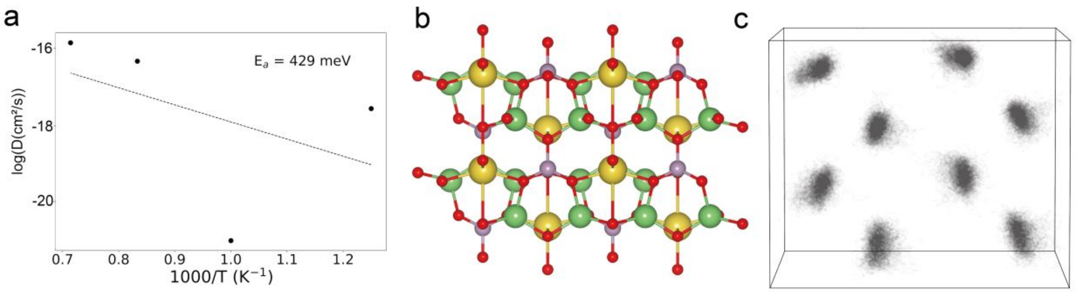

Figure S8. AIMD simulations of $NaLi_2PO_4$. a) Arrhenius plot of lithium-ion diffusion coefficient. b) The structure schematic. c) Isosurface of lithium-ion probability density from the 800-1400K AIMD simulation.

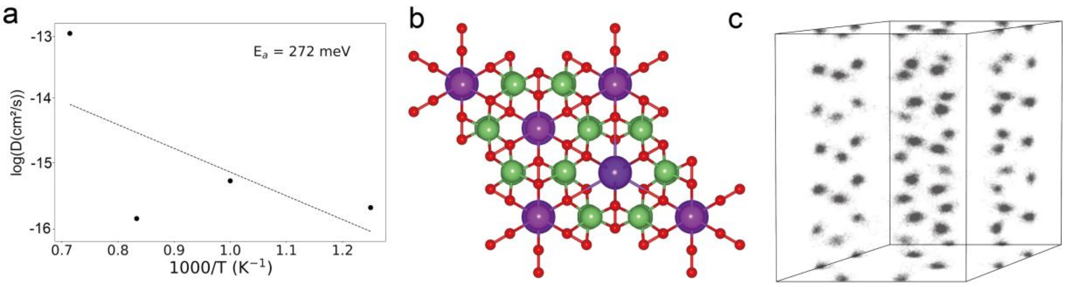

Figure S9. AIMD simulations of KLi_6BiO_6. a) Arrhenius plot of lithium-ion diffusion coefficient. b) The structure schematic. c) Isosurface of lithium-ion probability density from the 800-1400K AIMD simulation.

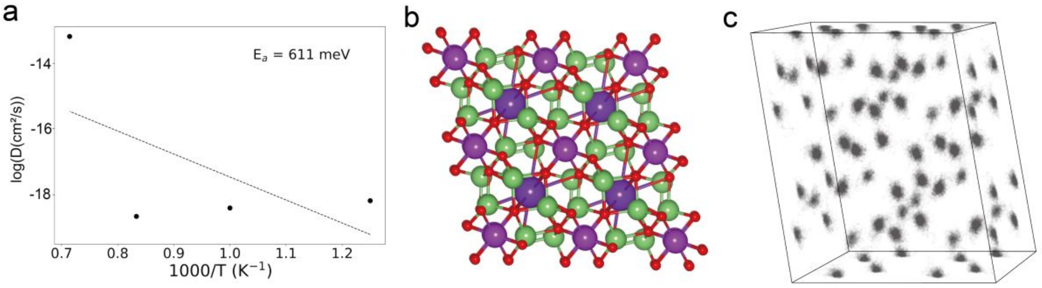

Figure S10. AIMD simulations of Li_6KBiO_6. a) Arrhenius plot of lithium-ion diffusion coefficient. b) The structure schematic. c) Isosurface of lithium-ion probability density from the 800-1400K AIMD simulation.

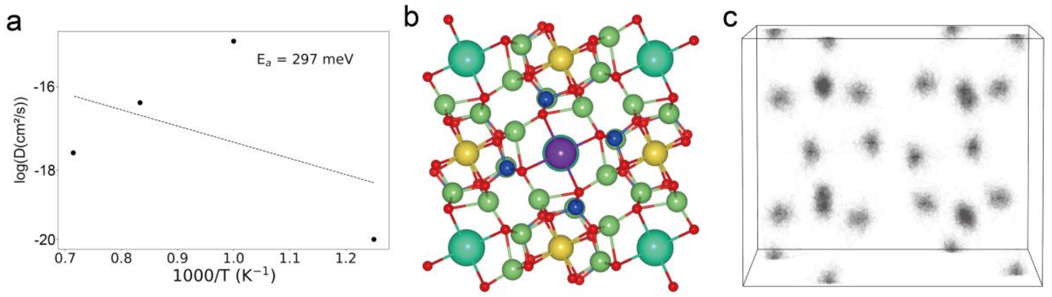

Figure S11. AIMD simulations of $CsKNa_2Li_8(Li(SiO_4))_4$. a) Arrhenius plot of lithium-ion diffusion coefficient. b) The structure schematic. c) Isosurface of lithium-ion probability density from the 800-1400K AIMD simulation.

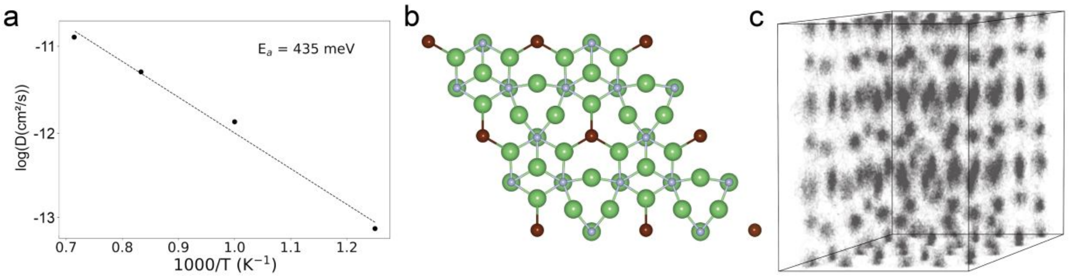

Figure S12. AIMD simulations of $Li_{10}N_3Br$. a) Arrhenius plot of lithium-ion diffusion coefficient. b) The structure schematic. c) Isosurface of lithium-ion probability density from the 800-1400K AIMD simulation.

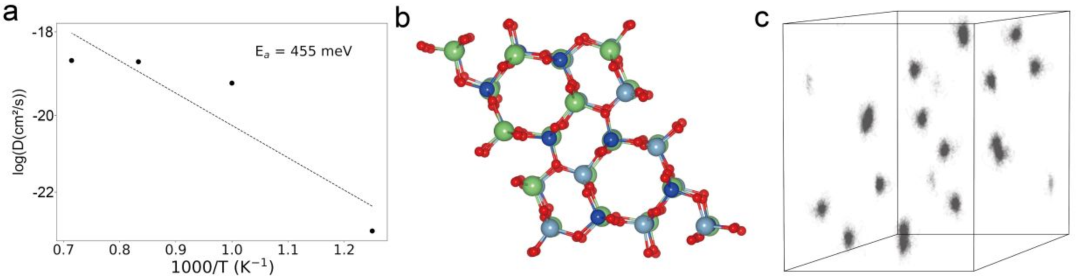

Figure S13. AIMD simulations of $LiAlSiO_4$. a) Arrhenius plot of lithium-ion diffusion coefficient. b) The structure schematic. c) Isosurface of lithium-ion probability density from the 800-1400K AIMD simulation.

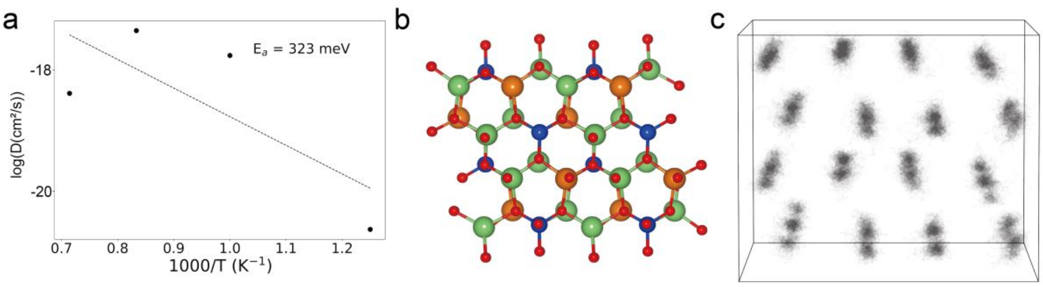

Figure S14. AIMD simulations of Li_2MgSiO_4. a) Arrhenius plot of lithium-ion diffusion coefficient. b) The structure schematic. c) Isosurface of lithium-ion probability density from the 800-1400K AIMD simulation.

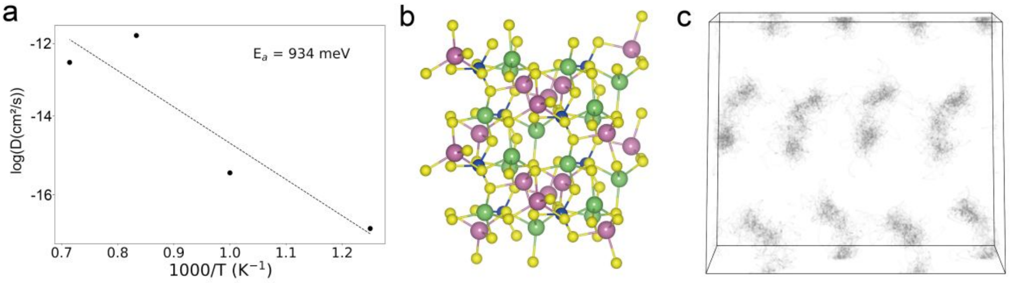

Figure S15. AIMD simulations of $In_2Li_2SiS_6$. a) Arrhenius plot of lithium-ion diffusion coefficient. b) The structure schematic. c) Isosurface of lithium-ion probability density from the 800-1400K AIMD simulation.

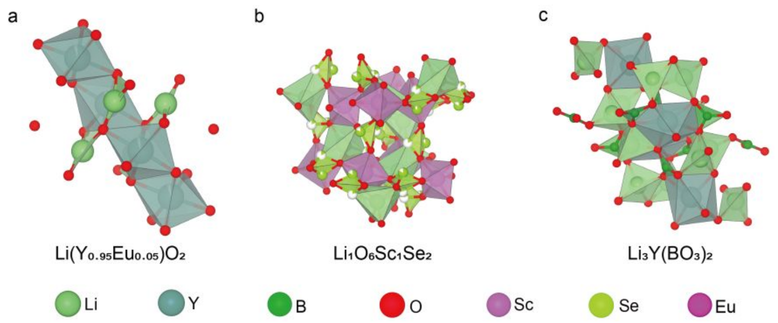

Figure S16. Figures a–c show representative structures of Li-containing compounds with transition metals that were excluded from our screening.

As part of our manual filtering process, we identified Li-containing structures that were excluded due to the presence of transition metals. Examples are shown in Figure R1. Specifically, structures FigureS16a and FigureS16c contain yttrium (Y), a relatively large and electropositive cation that tends to form highly stable coordination environments, potentially hindering the formation of a percolating Li-ion conduction network. FigureS16b includes both scandium (Sc) and selenium (Se); Sc often forms compact, rigid frameworks with limited Li mobility, while Se, being a heavier and more polarizable anion than O or S, may contribute to structural softness or instability under operating conditions. These factors collectively result in poor Li-ion connectivity in these materials. All related structures and explanations have been added to the Supporting Information.
